# Behavioral Impact of the Regulation of the Brain 2-Oxoglutarate Dehydrogenase Complex by Synthetic Phosphonate Analog of 2-Oxoglutarate: Implications into the Role of the Complex in Neurodegenerative Diseases

**DOI:** 10.4061/2010/749061

**Published:** 2010-10-26

**Authors:** L. Trofimova, M. Lovat, A. Groznaya, E. Efimova, T. Dunaeva, M. Maslova, A. Graf, V. Bunik

**Affiliations:** ^1^Departments of Biophysics, Biology Faculty, Lomonosov Moscow State University, Moscow 119992, Russia; ^2^Departments of Physiology, Biology Faculty, Lomonosov Moscow State University, Moscow 119992, Russia; ^3^Belozersky Institute of Physico-Chemical Biology, Lomonosov Moscow State University, Moscow 119992, Russia

## Abstract

Decreased activity of the mitochondrial 2-oxoglutarate dehydrogenase complex (OGDHC) in brain accompanies neurodegenerative diseases. To reveal molecular mechanisms of this association, we treated rats with a specific inhibitor of OGDHC, succinyl phosphonate, or exposed them to hypoxic stress. In males treated with succinyl phosphonate and in pregnancy-sensitized females experiencing acute hypobaric hypoxia, we revealed upregulation of brain OGDHC (within 24 hours), with the activity increase presumably representing the compensatory response of brain to the OGDHC inhibition. This up-regulation of brain OGDHC was accompanied by an increase in exploratory activity and a decrease in anxiety of the experimental animals. Remarkably, the hypoxia-induced elevation of brain OGDHC and most of the associated behavioral changes were abrogated by succinyl phosphonate. The antagonistic action of hypoxia and succinyl phosphonate demonstrates potential therapeutic significance of the OGDHC regulation by the phosphonate analogs of 2-oxoglutarate.

## 1. Introduction

A number of inborn [1–5] and acquired [6–13] neuropathologies are associated with impaired function of the mitochondrial 2-oxoglutarate dehydrogenase multienzyme complex (OGDHC). OGDHC comprises multiple copies of the three catalytic components: the thiamine diphosphate-dependent 2-oxoglutarate dehydrogenase (E1o), the lipoyl-bearing dihydrolipoamide succinyl transferase (E2o), and the FAD-binding dihydrolipoamide dehydrogenase (E3). Coupled action of the components is required for an important regulatory step in the mitochondrial tricarboxylic acid cycle, the oxidative decarboxylation of 2-oxoglutarate (Reaction 1, where R = –CH_2_–CH_2_–COOH) generating energy in the form of NADH, and macroergic compound succinyl-CoA [14]:
(1)
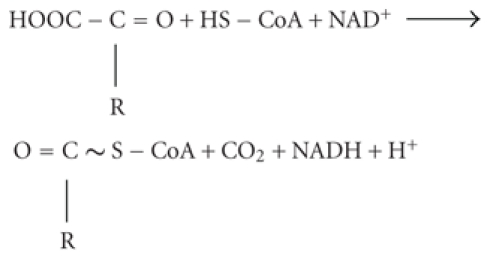



 To reveal molecular mechanisms of the association between neuropathologies and OGDHC function, we introduced specific inhibitors of OGDHC [15], which have been successfully applied in recent years for cellular [16–19], tissue [20], and animal [21] studies. Having the phosphonate residue instead of the leaving carboxyl group of 2-oxoglutarate, these synthetic inhibitors target the starting and rate-limiting E1o component of OGDHC in a highly specific manner, imitating transition state of the E1o-catalyzed step [15, 22]. Hence, application of such phosphonate analogs of 2-oxoglutarate allows modeling the states when a decrease in the OGDHC activity is observed. In particular, such a decrease occurs in brains of patients with neurodegenerative diseases, including Alzheimer's [9, 10, 13] and Parkinson's [6, 11] diseases, Wernicke-Korsakoff syndrome [12], and progressive supranuclear palsy [8]. It is important to note that 2-oxoglutarate is the glutamate precursor in the glutamate synthesis from glucose *de novo*. In view of this, the irreversible degradation of 2-oxoglutarate by OGDHC (Reaction 1) is intimately related to the synthesis/degradation of excitatory (glutamate) and inhibitory (GABA) neurotransmitters. Indeed, the perturbed flux through the complex was shown to affect the amino acid levels [23], which may explain the developmental impact of the OGDHC regulation, shown in our previous paper [21]. In the present work, we apply the OGDHC inhibitor succinyl phosphonate to study the behavioral impact of the OGDHC function in adult rats. We show a compensatory response of cortex and striatum to the OGDHC inhibition, which correlates with behavioral changes. Increasing the inhibition or combining the inhibition with hypoxia may abrogate the response. The antagonistic action on OGDHC of the hypoxic stress and synthetic inhibitor is of potential therapeutic significance.

## 2. Methods

### 2.1. Animal Experiments

All experiments were performed with consent to Helsinki Declaration on the Guide for the Care and Use of Laboratory Animals, defining the conduct of ethical research on laboratory and other animals. Animals were kept at 21 ± 2°C on standard ration and 12/12 h light/dark cycle. Wistar rats of about 200 g (males) or 250–300 g (females) were used in the experiment. Pregnant rats were exposed to hypobaric hypoxia at day 9–10 of pregnancy by placing in a decompression (altitude) chamber of 3.3 L volume, with a vacuum pump “Mez Mohelnice” (Mohelnice, Czech Republic). Acute hypoxia was achieved by decreasing the atmospheric pressure in 1 min to 145 mm Hg, correspondent to 11500 m altitude [24]. SP was introduced to animals at 5 and 25 mg/kg by intranasal application of the water solution of the trisodium salt, with the physiologic solution substituting for SP in all reference groups. In the study of the SP influence on hypoxic effects, the compound was introduced 45–50 min before hypoxic treatment. Experimental groups comprised 6–15 animals.

### 2.2. Behavioral Parameters

They were estimated in the standard tests: open-field [25], elevated plus maze [26]; light-dark chamber [27], and closed plus maze [28]. Video-recording and ≪Easy Track≫ program [29] were used to follow behavior. To increase the assessment power, the data from different behavioral tests were analyzed. Different tests were also employed to exclude the animal adaptation to experimental conditions when the time dependence of the behavioral changes was studied. The parameters presented in figures correspond to those which showed statistically significant changes (*P* < .05) and the changes at the level of trends (*P* < .1). The latter provided additional support for the statistically significant changes, thus increasing the conclusion accuracy.

### 2.3. Tissue Samples and Enzyme Assay

After physiological monitoring has been performed, the animals were sacrificed by decapitation; cortex and striatum were excised on ice quickly, frozen in liquid nitrogen, and stored at −70°C until assay. OGDHC activity was extracted and assayed as described earlier [21]. Each sample corresponded to one animal, with the OGDHC assay in a sample repeated 3-4 times at three different protein concentrations. This was done to ensure that the activity is estimated in the interval where the dependence of the reaction rate on the catalyst concentration is linear. Preservation of the linearity at low and high protein concentrations attests to the absence of the multienzyme complex dissociation upon dilution and of the interfering activities consuming the produced NADH, respectively. The OGDHC activity is expressed as *μ*mol/min per g of wet tissue.

### 2.4. Reagents

Succinyl phosphonate was synthesized and purified according to published conditions [15]. Halt Protease Inhibitor Single-Use Cocktail was from “Pierce;” CoA was from “Gerbu”, Germany, and other chemicals were from “Sigma,” USA. 

#### 2.4.1. Statistical Analysis

It was performed using Statistica 6.0 Software, Inc.. Values are expressed as means ± SEM. Dispersion of the ratios of the affected to control values (%) was calculated by taking into account experimental errors in determination of both values as in [30]. Statistical significance of the differences in the parameter mean values was tested by one-way analysis of variance (ANOVA) followed the Student-Newman-Keuls post hoc test. Statistical significance of the differences in the animal stratification by the resistance to hypoxia was assessed by the Fisher's exact test.

## 3. Results

### 3.1. OGDHC Inhibitor SP Induces Behavioral Changes Concomitantly with Up-Regulation of the Brain OGDHC in Male Rats

**Figure 1:**
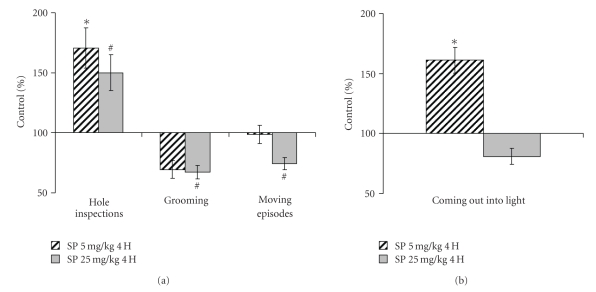
Changed behavioral parameters of male rats tested in the open field (a) and elevated plus maze (b) 4 hours after the treatment with 5 and 25 mg/kg SP. Statistical significance: *P < .05; ^#^ *P* ≤ .1.

**Figure 2:**
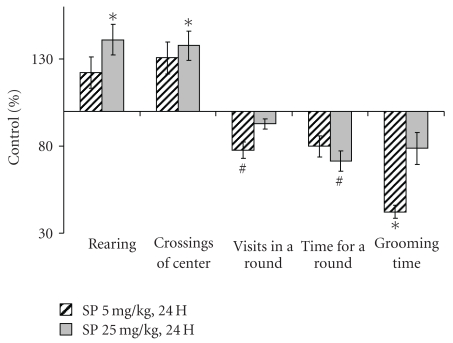
Changed behavioral parameters of male rats tested in the closed plus maze 24 hours after the treatment with 5 and 25 mg/kg SP. Statistical significance: *P < .05; ^#^ *P* ≤ .1.

Behavior was assessed within 4 h (Figure 1) and the next day (Figure 2) after application of SP. All together, the results of the open field, elevated plus maze and closed plus maze tests pointed to increased exploratory activity and decreased anxiety, resulting from the SP application. An increase in exploratory activity was obvious from statistically significant elevations in the hole inspections (Figure 1(a)), coming out into light (Figure 1(b)), rearing and crossing the center (Figure 2). Visits and time per a round in the closed plus maze showed a trend to decrease (Figure 2), suggesting improved orientation ability and inspection efficiency [28]. Along with the mentioned above increase in the coming out into light (Figure 1), the shorter grooming times (Figures 1(a) and 2) pointed to decreased anxiety. It is noteworthy, that these effects were not pronounced when the SP-treated animals were imposed to stress. That is, under stressful conditions of the light-dark chamber, no statistically significant changes in the exploratory activity and anxiety were detected 24 h after the treatment (data not shown). Thus, the SP-induced increases in exploratory activity and risk behavior do not persist under potentially dangerous conditions when animal is challenged with a bright light. The difference of the animal response to the SP treatment, revealed under comfortable and stressful conditions, is indicative of a certain adequacy of the response, supporting its behavioral rather than motoric origin.

Remarkably, increasing SP to 25 mg/kg did not always increase the behavioral effect of 5 mg/kg SP, often even alleviating effects of the low dose (Figures 1 and 2). At the same time, elevating SP tended to decrease the moving episodes (Figure 1(a)) and muscle force (data not shown). It thus appears that at 25 mg/kg the systemic action of SP increases, with the muscle OGDHC inhibition obviously causing energetic impairment and muscular weakness. This may complicate interpretation of behavioral changes assessed by the animal movements. Thus, there is a specific window of the SP doze where the OGDHC inhibitor increases the exploratory activity and decreases anxiety of experimental animals. 

**Figure 3:**
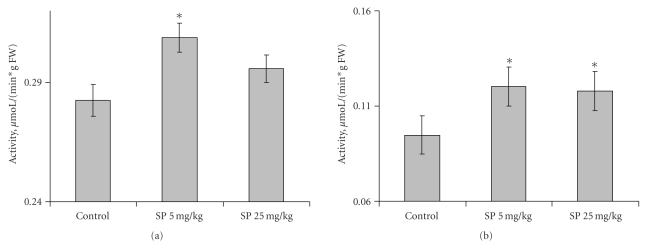
Influence of the SP treatment (5 and 25 mg/kg) on the cortex (a) and striatum (b) activity of OGDHC in male rats. Statistical significance: *P < .05.

After the behavioral parameters were assessed, the animals were sacrificed and the OGDHC activity in the extracts of cortex and striatum determined (Figure 3). The cortex activity was increased at 5 mg/kg SP, returning to the control level at 25 mg/kg SP (Figure 3(a)). SP also increased the OGDHC activity in striatum (Figure 3(b)), with the maximal effect achieved already at a low doze. Thus, increases in the OGDHC activity of cortex and striatum correlate with the increased exploratory activity and decreased anxiety of the animals, as observed under comfortable conditions in the open field, elevated plus maze and closed plus maze tests.

### 3.2. Protective Action of the SP Preconditioning upon Acute Hypoxia

**Figure 4:**
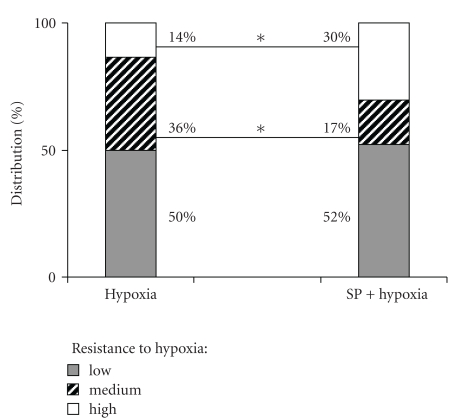
Influence of the pre-treatment with 5 mg/kg SP on the resistance of pregnant rats to acute hypobaric hypoxia. Statistical significance: *P < .05.

In neurodegenerative diseases, the metabolic stress is often increased by hypoxia, in which damaging action is associated with the elevated ROS and glutamate excitotoxicity. In cellular experiments, SP was shown to protect from the glutamate-induced ROS [18] and excitotoxicity [16, 17, 21]. Owing to this, we tested if SP would show a protective action on the behavioral and biochemical changes induced by hypoxia. We used the previously established model, in which acute hypoxia was created in a decompression chamber with female rats sensitized to the insult by pregnancy [21, 24, 31]. Protective action of SP was observed already during the acute hypoxia in decompression chamber. Within an animal group, the resistance to hypoxia assessed by both physiological and behavioral parameters is known to vary, defined as low, medium, or high when the time before collapsing under hypoxic conditions is less than 5 minutes, between 5 and 10 minutes, and 10 minutes and more, respectively [24, 32].  Figure 4 shows that pre-conditioning with a low doze of SP (5 mg/kg) increased the percentage of the highly resistant animals at the expense of the medium resistant group. The SP-induced increase in the number of animals highly resistant to hypoxic conditions exhibits the protective effect of the pretreatment with the OGDHC effector upon acute hypoxia.

**Figure 5:**
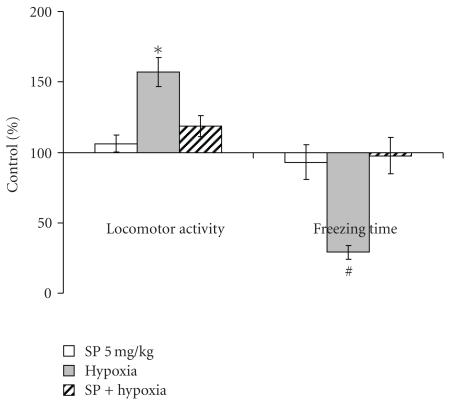
Influence of the pre-treatment with 5 mg/kg SP on the behavioral effects of the acute hypobaric hypoxia of pregnant rats with low resistance to hypoxia in the open field test 24 hours after hypoxia. Statistical significance: *P < .05; ^#^ *P* ≤ .1.

**Figure 6:**
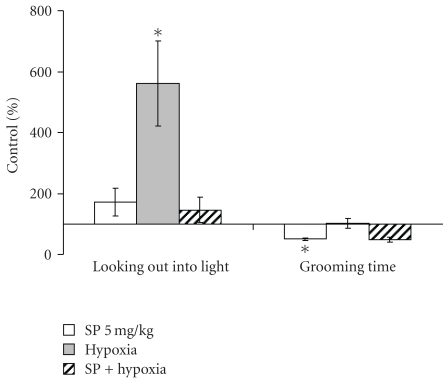
Influence of the pre-treatment with 5 mg/kg SP on the behavioral effects of the acute hypobaric hypoxia of pregnant rats with low resistance to hypoxia in the elevated plus maze test 24 hours after hypoxia. Statistical significance: *P < .05.

**Figure 7:**
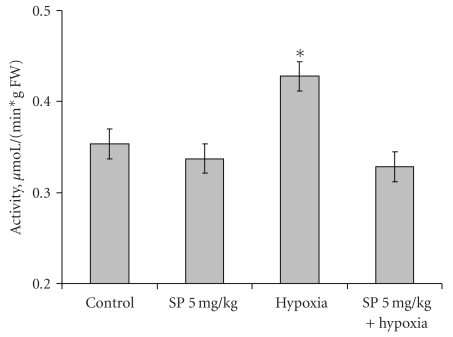
Influence of 5 mg/kg SP on the elevation in the cortex activity of OGDHC, induced by acute hypoxia in pregnant rats. Statistical significance: *P < .05.

The behavioral effects were determined in the most reactive low resistant rats 24 hours after recovery from the hypoxia-induced collapse. Compared to the males assessed at the same time after the SP treatment (Figure 2), pregnant females showed more resistance to the action of SP *per se* (Figures 5 and 6). That is, SP alone did not significantly influence the behavioral parameters in the open field or elevated plus maze tests. Only the grooming time was significantly decreased by the low SP doze in both females (2-fold, Figure 6) and males (3-fold, Figure 2), indicative of the sex-independent anxiolytic effect of SP on animals. Remarkably, not only SP, but also hypoxia *per se* showed an anxiolytic effect. However, the latter was expressed in the rat sensitivity to light rather than grooming. That is, hypoxia caused the statistically significant increase in the looking out into light (Figure 6), with the freezing time tending to decrease (Figure 5), but did not change the grooming time (Figure 6). Thus, a certain degree of similarity in the action of both SP and hypoxia was revealed, although the common anxiolytic effect was expressed in different behavioral parameters, such as grooming or light sensitivity in the SP or hypoxia treatments, respectively. Noteworthy, the SP pre-treatment abrogated the hypoxia effects, exposing the protective action of SP upon hypoxia. That is, the hypoxia-induced changes in the locomotor activity and freezing time were abolished in the SP-treated animals (Figure 5), as was the statistically significant increase in the looking out into light (Figure 6). Analysis of the OGDHC activity in cortex of pregnant females provided further evidence on the SP-induced protection from the hypoxic effects. As seen from Figure 7, hypoxia increased the OGDHC activity in cortex, whereas pre-treatment with SP returned the OGDHC activity to the control level. The data of Figure 7 also support the conclusion that the OGDHC activity level correlates with behavioral parameters. That is, the hypoxia-induced behavioral effects (Figures 5 and 6) were accompanied by the up-regulation of the cortex OGDHC, with the combined action of hypoxia and SP returning both the OGDHC activity (Figure 7) and behavioral parameters (Figures 5 and 6) close to the norm.

Thus, the pre-treatment with a low SP doze (5 mg/kg) normalized the hypoxia-induced changes in the cortex OGDHC activity (Figure 7) and behavioral parameters (Figures 5 and 6), increasing the proportion of rats exhibiting the high resistance to hypoxia (Figure 4). This means that the SP pre-treatment protected from hypoxia on biochemical, behavioral, and physiological levels.

## 4. Discussion

In male rats treated with the OGDHC inhibitor SP, we revealed increased exploratory activity and decreased anxiety (Figures 1 and 2) concomitant with increases in the OGDHC activity in cortex and striatum (Figure 3). This result complements the known correlation found in human subjects. That is, the impaired cognition in patients with neurodegenerative diseases was observed along with the decreased OGDHC activity in their brains [7], whereas in animal model we observe increased exploratory activity with the orientation and inspection efficiency tending to improve (Figures 1 and 2), when the brain OGDHC activity is elevated (Figure 3).

It should be kept in mind that the OGDHC activity assayed in brain extracts under standard test conditions is not equal to the flux through OGDHC inside brain cells. The flux may differ dependent on the cell type, being determined, in particular, by the substrate concentrations within the cellular mitochondria, which are most probably different from those in the standard assay system *in vitro*. However, the activity assays do show that (i) application of SP induces the brain response at the level of the OGDHC regulation, and (ii) the response is to up-regulate OGDHC, obviously compensating for the enzyme inhibition by SP. The compensatory response of brain to the OGDHC inhibition by SP (Figure 3) may occur through activation of internal mechanisms of the OGDHC stimulation, for example, by protein-protein interactions, posttranslational modifications, increased synthesis. In particular, increased synthesis of the first component of OGDHC, E1o, was observed in response to the acute ethanol-induced stress [33]. Besides, in addition to its action as the reversible OGDHC inhibitor [15], SP is a protector of OGDHC from an irreversible inactivation occurring in the course of catalysis [16, 17]. During a certain time period, this protective effect may manifest itself as an apparent increase in the OGDHC activity compared to the control value with the unprotected enzyme (Figure 3). It cannot, however, be the only reason for the increase, as the latter may be observed in the absence of SP as well. That is, the OGDHC activity of cortex increases also as a result of acute hypoxia in pregnant rats (Figure 7). Worth noting, also in this case the elevation of the OGDHC activity in cortex is accompanied by increased exploratory activity and decreased anxiety (Figures 5 and 6). 

In our earlier work on the developmental impact of the brain OGDHC activity, we observed the sex-determined differences in the brain OGDHC expression and reactivity to SP [21]. The present study confirms dependence of the behavioral impact of the OGDHC regulation on the animal physiology, as behavior of pregnant females is less responsive to SP than that of males. Nevertheless, the sex-independent anxiolytic effect of SP was also observed, with the low doze of SP decreasing the grooming time in both males (Figures 1(a) and 2) and females (Figure 6). 

Remarkably, both earlier [21] and here, the SP action exhibited an interplay with different stresses. For instance, in males, the SP-induced increase in exploratory activity and decrease in anxiety were pronounced only under comfortable test conditions (Figures 1 and 2), but not under the stress conditions of the light-dark chamber (data not shown). Likewise, in pregnant rats exposed to hypoxia, the SP pre-treatment abrogated most of the hypoxia-induced behavioral changes (Figures 5 and 6). Combination of the hypoxic stress with SP also reduced the brain OGDHC activity elevated by hypoxia (Figure 7). Our data thus point to the antagonistic action of stress and SP at both biochemical and behavioral levels. This antagonism could be used to protect from the negative effects of the metabolic stress. In particular, we show the protection in the hypoxic model, where SP not only normalized the behavioral parameters (Figures 5 and 6) and OGDHC activity in cortex (Figure 7), but also increased the proportion of rats highly resistant to hypoxia (Figure 4).

Earlier, we suggested that metabolic stress accompanying the onset of neurodegeneration increases an irreversible inactivation of OGDHC [34, 35], which may also lead in appearance of aberrant enzyme forms with an elevated ability to catalyze hazardous side reactions [36]. This irreversible damage of the OGDHC function occurring in neurodegenerative diseases [7, 36] may be alleviated through the reversible inhibition of OGDHC by SP due to several reasons. First of all, the inhibition by SP may induce compensatory effects up-regulating OGDHC (Figure 3). Furthermore, as shown earlier, SP binding to the active site protects the essential groups of enzyme from irreversible modifications causing inactivation [16, 17]. The SP binding also prevents the enzyme from catalysis of the side reactions, including production of ROS and enzyme-bound radicals irreversibly damaging OGDHC [18]. The work presented here extends our previous *in vitro* and *in situ* studies to animal experiments, showing that reversible inhibition of OGDHC under conditions of metabolic stress may indeed normalize both the behavior (Figures 5 and 6) and brain OGDHC activity level (Figure 7). It is probable that up-regulation of OGDHC occurs also in response to its irreversible inactivation upon initial stages of the neurodegenerative diseases. However, when the inactivatory conditions persist with the up-regulated OGDHC not protected, the cellular compensatory ability is exhausted, eventually resulting in the increasing damage of the brain mitochondrial metabolism. In contrast, the protective effects of SP described above would allow cells to combine the OGDHC up-regulation with the enzyme protection, which may underlie cellular ability to overcome metabolic stress.

## 5. Conclusions

Up-regulation of the brain OGDHC activity correlates with increased exploratory activity and decreased anxiety. Compensatory response of brain to metabolic stress may include up-regulation of OGDHC. The up-regulation under metabolic stress may be adjusted by reversible inhibition of OGDHC, normalizing the biochemical and behavioral deviations.

## Abbreviations

E1o: 2-oxoglutarate dehydrogenase;
E2o: Dhydrolipoamide succinyl transferase;
E3: Dihydrolipoamide dehydrogenase;
OGDHC: 2-oxoglutarate dehydrogenase complex;
ROS: Reactive oxygen species;
SP: Succinyl phosphonate.
